# Effects of Grain Source and Emulsifier Supplementation of Reduced-Energy Diets on Performance, Slaughter Traits, Tibia Characteristics, Jejunal Histomorphology and Pancreatic Enzymes in Broilers

**DOI:** 10.3390/ani16203269

**Published:** 2026-10-16

**Authors:** Osman Olgun, Yusuf Cufadar, Seyit Ahmet Gökmen, Esra Tuğçe Gül, Ahmet Engin Tüzün, Bilal Selçuk

**Affiliations:** 1 Department of Animal Science, Faculty of Agriculture, Selçuk University, 42130 Konya, Türkiye; oolgun@selcuk.edu.tr (O.O.); ycufadar@selcuk.edu.tr (Y.C.); esra.gul@selcuk.edu.tr (E.T.G.); 2 Department of Plant and Animal Production, Lâdik Vocational School, Ondokuz Mayıs University, 55500 Samsun, Türkiye; bilal.selcuk@omu.edu.tr; 3 Kocarlı Vocational School, Adnan Menderes University, 09970 Aydın, Türkiye; atuzun@adu.edu.tr

**Keywords:** barley, emulsifier, histomorphology, pancreatic enzymes, tibia

## Abstract

The purpose of this study was to evaluate the effect of emulsifier addition to reduced-energy maize-, barley- or wheat-based diets on selected indicators in broilers. In this experiment, a total of 540 unsexed (mixed-sex) one-day-old Ross 308 chicks were allocated to six treatment groups using a 3 × 2 factorial design (five replicate pens of 18 birds). Each diet was fed either as a standard-energy diet without emulsifier or as a reduced-energy diet (−50 kcal/kg ME, achieved by lowering soybean oil inclusion) supplemented with 500 g/ton emulsifier. The wheat-based diet reduced body weight and feed consumption but improved feed conversion ratio compared with the barley-based diet. Feeding a maize-based diet increased carcass yield and the breast (meat, skin and bone) ratio but decreased the leg (two thighs and two drumsticks, with meat, skin and bone) ratio compared to feeding a wheat-based diet. The addition of emulsifiers to barley- and wheat-based diets increased carcass yield. The negative effects of barley- and wheat-based diets on the carcass compared to the maize diet were compensated for by the addition of emulsifiers to the diet. The highest values for tibia breaking strength and tibia ash content were obtained with wheat-, maize-, and barley-based diets, respectively. Tibia ash increased with the addition of emulsifiers to barley- and wheat-based diets. Barley- and wheat-based diets resulted in higher pancreatic amylase activity compared to maize-based diets. Pancreatic lipase activity increased with the addition of emulsifiers. The wheat-based diet increased villus surface area compared to other cereals. The addition of emulsifier to the diet increased villus height/crypt depth but decreased other villus measurements. In conclusion, it can be said that adding emulsifiers to reduced-energy broiler diets feed is more effective in increasing carcass yield in barley- and wheat-based feeds, and that emulsifier addition compensates for the energy deficit in the diet.

## Simple Summary

Cereal grains such as maize, barley and wheat are the main energy source in chicken feeds. However, barley and wheat are harder for young birds to digest and often need extra oil to match the energy of maize. Emulsifiers are feed additives that help birds make better use of dietary fat. In this study we fed broiler chickens diets based on maize, barley or wheat, each with or without an emulsifier, and measured how well the birds grew, their meat yield, bone strength, gut structure and digestive enzymes. Birds fed the wheat-based diet consumed significantly less feed and had a lower body weight, while the barley and wheat diets reduced carcass yield. In the emulsifier treatments, dietary energy was reduced by lowering the amount of added soybean oil. Compared with the standard-energy diets, these emulsifier-supplemented reduced-energy diets supported similar growth, restored carcass yield and increased tibia ash content in the barley and wheat diets, and increased the activity of the fat-digesting enzyme lipase. These results suggest that emulsifier supplementation of reduced-energy diets is a useful feeding strategy, particularly when maize is replaced by barley or wheat, as it may reduce feed costs without impairing growth.

## 1. Introduction

Maize is commonly the main cereal used in broiler diets because it provides a high level of available energy and, unlike wheat and barley, contains relatively low levels of soluble non-starch polysaccharide (NSP) that do not increase intestinal viscosity [1,2]. However, maize requires large amounts of water and can only be cultivated in limited areas. For these reasons, replacing maize with more conventional energy sources such as barley and wheat is occasionally considered. Although barley and wheat contain fewer calories than maize, they are richer in protein and amino acids [3]. Nevertheless, unlike maize, barley and wheat are abundant in water-soluble NSPs such as arabinoxylans and beta-glucans, which increase intestinal viscosity and hinder nutrient absorption and digestion [4,5,6]. Consequently, higher levels of dietary oil are required to balance the energy content of these diets.

Young birds have limited fat digestion capacity because they secrete insufficient bile and lipase [7]. One strategy to improve fat digestibility, particularly in chicks, is the use of exogenous emulsifiers, which lower the surface tension of lipids and allow immiscible compounds to mix more readily [8]. Since lipase and bile secretions are inadequate in young birds, emulsifier supplementation enhances fat digestibility [9]. Recent studies indicate that emulsifiers, particularly monoglyceride-based additives, can improve lipid digestion and thereby indirectly support mineral absorption [8,10]. Additionally, fatty acids can saponify with calcium to form insoluble calcium soaps, which are unavailable to the bird and may lead to calcium deficiency, impairing bone development and causing skeletal disorders [11,12]. Therefore, emulsifiers can enhance the efficient utilization of dietary fat sources, enabling the formulation of lower-energy diets and reducing feed costs without compromising broiler growth. As a result, emulsifiers have the potential to minimize economic losses by preventing growth retardation in broilers.

When emulsifiers are used in combination with NSP-degrading enzymes and multi-enzymes, their effectiveness is further enhanced [13,14,15]. This suggests that NSPs may restrict the functional capacity of emulsifiers. Furthermore, the inclusion of grains other than maize in broiler diets has been shown to increase dietary crude fat levels to meet energy requirements [16]. Therefore, the hypothesis of this study is that emulsifier supplementation would allow a 50 kcal/kg reduction in dietary ME without impairing broiler performance, and that this response would differ depending on the grain source. The aim of this study was to determine the effect of emulsifier supplementation on performance, carcass characteristics, tibia parameters, jejunum histomorphology, and pancreatic enzyme secretion in broilers fed corn-, barley-, or wheat-based diets and their low-energy counterparts.

## 2. Materials and Methods

### 2.1. Birds and House Management

To investigate the effects of six distinct nutritional regimens, a 42-day trial was carried out utilizing 540 one-day-old, unsexed Ross 308 chicks at the Faculty of Agriculture, Selçuk University (Konya, Türkiye). These chicks, obtained from a local supplier, were placed in 30 floor units (1.5 × 1.5 m) in a completely randomized design, in treatment groups consisting of six different diets, with five replicates and 18 chicks in each subgroup. The sample size was determined based on previous comparable trials [17,18,19], which demonstrated adequate statistical power for detecting treatment differences of ≥5% in productive traits. Environmental conditions were strictly governed by an automated aeration system managing ventilation, heating, and cooling, with wood shavings utilized as litter material over the floor pens. Feed and water were available ad libitum for 24 h/day throughout the trial via feeders (round hanging type) and nipple drinkers in each pen, and the drinking water was tap water supplied at approximately 15–18 °C. A temperature of 33 °C was maintained from days 1 to 7, followed by a gradual reduction to 21–23 °C for the remainder of the rearing period. The relative humidity in the house ranged from 50 to 70% throughout the trial. The lighting schedule was systematically altered: the birds received a 23:1 h light-to-dark photoperiod during the first week, which was modified to an 18:6 h cycle for the subsequent weeks. The lighting program was set up using white fluorescent lamps to provide an illuminance of 15 lx. Flocks were monitored daily to track overall health and record any incidence of mortality.

### 2.2. Experimental Design

The compositions of the experimental diets based on maize, barley, and wheat are given in Table 1, Table 2, and Table 3, respectively. The maize, barley, and wheat used in the rations were sourced from a domestic company. All experimental rations were prepared and offered in mash form. For each grain source, two diets were prepared: a standard-energy diet without emulsifier and a reduced-energy diet supplemented with 500 g/ton of an emulsifier (containing 55% hydrolyzed lecithin on a silicate carrier and a minimum of 8% lysophospholipids). These six diets, consisting of three grain sources and two dietary treatments (standard-energy diet without emulsifier and reduced-energy diet with emulsifier), were tested in 30 pens of 18 birds each, using a 3 × 2 factorial arrangement in a completely randomized design with five replicates. A total of 540 Ross 308 broiler chicks were used in the experiment, with 90 unsexed day-old chicks assigned to each treatment group. The experimental diets were prepared according to Aviagen’s [20] recommended dietary requirements for chicks at each period. Diets containing emulsifiers were formulated to have energy levels 50 kcal/kg ME lower than those without. This reduction was achieved by decreasing soybean oil inclusion by 8.5–10 g/kg (Table 1, Table 2 and Table 3). This design was chosen deliberately to reflect the energy-sparing approach used in commercial practice: the diets without emulsifier served as standard-energy (positive control) diets, and the aim was to determine whether the reduced-energy diets supplemented with emulsifier could achieve the same results.

### 2.3. Determination of Performance Parameters

The body weight of the chicks was determined by weighing all the birds in each subgroup at hatching and on the last day of the experiment (day 42), and the average was calculated as g/bird. Body weight gain was calculated from these data. Experimental feeds were given to each subgroup by weight, the feed remaining in the feeder at the end of the experiment was weighed, and feed consumption was calculated from these data as g/bird. Feed conversion ratio was calculated as g feed/g gain with feed consumption/body weight gain formula. All weighings of birds and feed were performed using a digital scale (Desis B5, DESİS, Tekirdağ, Türkiye) with a readability of 1 g.

### 2.4. Determination of Slaughtering Traits

At the end of the experiment (day 42), all birds in each pen were weighed, and two unsexed birds with a body weight close to the pen average were selected from each pen (60 birds in total). The selected birds were individually weighed and transferred to the slaughter unit of the Faculty of Agriculture, where they were stunned by a percussive blow to the head and killed by exsanguination through a ventral neck cut. After a bleeding period of 5 min, death was confirmed by an experienced researcher on the basis of the absence of heartbeat, breathing and movement, after which cervical dislocation was performed. The carcass (eviscerated, without head, feet and edible giblets) and the examined body parts were then weighed.

### 2.5. Determination of Tibia Characteristics

The left tibia of the same two birds selected from each pen were collected to determine bone mechanical properties and, after cleaning, were stored in a freezer at −20 °C until analysis. The length and diameter of the tibias were measured with a caliper, and their weight was measured with a scale sensitive to 0.01 g. Afterwards, tibia breaking strength was determined as described by Wilson and Ruszler [21] and Gül et al. [22]. Tibia ash was determined in the right tibia of the same birds. Tibias were dried at 105 °C for 24 h, then weighed, ashed in a muffle furnace at 550 °C for 24 h and weighed again after ashing. Tibia ash content (%) was calculated by dividing the ash weight by the dry tibia weight and multiplying by 100.

### 2.6. Determination of Digestive Enzyme Activities

Enzyme activities were determined in the pancreas of two broilers per replicate, for a total of 60 birds. The pancreas was harvested from each broiler within 5 min after death, placed in aluminum foil, snap-frozen in liquid nitrogen, and stored at −80 °C. Enzyme extraction was performed according to the method described by Chen et al. [23]. Amylase activity was determined according to Bernfeld’s [24] method, chymotrypsin activity according to Hummel’s [25] report, and lipase activity according to the method of Sigurgísladóttir et al. [26].

### 2.7. Determination of Jejunum Histomorphological Parameters

Jejunum histomorphological parameters were measured using samples taken from two broilers per replicate, for a total of 60 birds. Samples were excised from the midpoint of the jejunum, between the point of entry of the bile ducts and Meckel’s diverticulum, as described by Xu et al. [27]. The samples taken for jejunum histomorphological measurements were immediately buffered in 10% formalin and gently shaken to remove adherent intestinal contents. Samples were kept in this solution for 72 h. After fixation, three cross-sections were obtained from each sample, processed using standard paraffin-embedding procedures and stained with hematoxylin and eosin [27]. Only intact and well-oriented crypt–villus units, in which the villus was sectioned longitudinally along its entire length from tip to base, were selected for measurement, and the mean value of the measurements was calculated for each bird [22,27]. Villus height (VH) was measured from the tip of the villus to the crypt–villus junction; villus width (VW) was measured at the midpoint of the villus between the brush borders of the opposing epithelial cells; and crypt depth (CD) was measured as the depth of the invagination between adjacent villi, down to the basement membrane of the crypt epithelial cells [22,27]. The VH/CD ratio was calculated by dividing VH by CD, and villus surface area (VSA) was calculated according to Sakamoto et al. [28].

### 2.8. Statistical Analysis

Data were analyzed using SPSS software (version 22.0; IBM Corp., Armonk, NY, USA). The Shapiro--Wilk test and Levene’s test were applied to verify the assumptions of normality and homogeneity of variances. In the statistical analysis, grain source and emulsifier supplementation (i.e., emulsifier-supplemented reduced-energy diet vs. standard-energy diet) were considered the main effects, and the mixed groups formed by these were considered the interaction effects. The pen was considered the experimental unit for all variables. For slaughter traits, tibia characteristics, pancreatic enzyme activities and jejunal histomorphology, the values of the two birds sampled from each pen were averaged, and the pen mean was used as a single observation in the statistical analysis (*n* = 5 pens per treatment). Results are presented as mean values accompanied by their respective standard errors (±SEM). Means were compared using Duncan’s multiple range test and differences were considered significant at *p* < 0.05.

## 3. Results

### 3.1. Performance

Table 4 lists the effects of treatments on broiler performance. The body weight (*p* < 0.01), body weight gain (*p* < 0.01), feed consumption (*p* < 0.01), and feed conversion ratio (*p* < 0.05) of broilers were all considerably affected by the use of different grains. The group fed a diet containing wheat had considerably lower body weight, body weight gain and feed consumption than the barley and maize groups, but these characteristics were statistically similar in the groups fed diets containing barley and maize (*p* < 0.01). The feed conversion ratio of the wheat group was statistically lower than that of the barley group, while being similar to that of the maize group (*p* < 0.05). Performance parameters did not differ between the emulsifier-supplemented reduced-energy diets and the standard-energy diets, and no grain × emulsifier interaction was observed (*p* > 0.05).

### 3.2. Slaughtering Traits

The effects of grain sources and emulsifier addition on the slaughtering traits of broilers are shown in Table 5. Carcass (% of body weight), breast (% of carcass), and legs (% of carcass) were statistically affected by the use of different grains in the diet (*p* < 0.01), but this effect was not observed in abdominal fat (% of body weight), liver (% of body weight) or gizzard (% of body weight) (*p* > 0.05). Carcass (% of body weight) and breast (% of carcass) were significantly higher in the maize group than in the groups fed diets containing barley and wheat (*p* < 0.01). The leg ratio was higher in the wheat group than in the maize group, while it was similar to the barley group (*p* < 0.01). Carcass yield (% of body weight) was higher in birds fed the emulsifier-supplemented reduced-energy diets than in those fed the standard-energy diets (*p* < 0.01). The other slaughter traits did not differ between these diets (*p* > 0.05). The grain × emulsifier interaction was significant only for carcass yield (% of body weight), which was lower in the barley and wheat groups without emulsifier than in the other groups (*p* < 0.01).

### 3.3. Tibia Traits

The effects of grain source and emulsifier supplementation on tibia characteristics are shown in Table 6. Grain source had no effect on the weight, length, diameter or wall thickness of the tibia (*p* > 0.05), but the breaking strength and tibia ash were significantly affected by the source of grains in the diet (*p* < 0.01). As for the tibia breaking strength and ash content, the wheat group had the highest values, followed by the maize and barley groups. Emulsifier supplementation of the reduced-energy diets significantly affected tibia wall thickness and ash content (*p* < 0.01), but not the other tibia traits (*p* > 0.05). The addition of emulsifier to the diet resulted in a significant increase in tibia wall thickness and tibia ash content. The interaction between grain type and emulsifier was found to have a significant effect on tibia wall thickness and tibia ash content (*p* < 0.01), but was not significant for other parameters (*p* > 0.05). Tibia wall thickness was found to be significantly higher in the wheat with emulsifier group than in the other interaction groups, and significantly lower in the wheat without emulsifier group than in the wheat with emulsifier and barley without emulsifier groups. The lowest tibia ash was obtained in the barley without emulsifier group, and the highest tibia ash was obtained in the wheat with emulsifier group.

### 3.4. Pancreatic Enzyme Levels

Table 7 lists the effects of different grain sources and emulsifier additions on pancreas (% of body weight) and pancreatic enzymes. Pancreas (% of body weight), pancreatic lipase, and protease activities were unaffected by the use of various grains in the diet (*p* > 0.05), but amylase activity was significantly affected by dietary grain source (*p* < 0.01). The barley and wheat groups significantly increased pancreatic amylase activity compared to the maize group (*p* < 0.05). Adding an emulsifier to the diet significantly increased pancreatic lipase activity (*p* < 0.01). Pancreatic amylase and protease activities did not significantly change with the addition of an emulsifier to the diet (*p* > 0.05). The interaction between grain source and emulsifier had a significant effect on pancreatic lipase activity (*p* < 0.01) but had no effect on other parameters (*p* > 0.05). It was observed that the addition of emulsifier significantly increased pancreatic lipase activity in the wheat group compared to the wheat and maize groups without emulsifier addition (*p* < 0.05); activity was similar to that in the other interaction groups.

### 3.5. Jejunum Histomorphology

The effects of the use of different grains and emulsifier addition on jejunum histomorphology are presented in Table 8. The grain source in the diet significantly affected all jejunum histomorphological parameters of broilers (*p* < 0.01). It was determined that the jejunum VH of the wheat group was substantially higher than that of the maize and barley groups, while it was significantly lower in the barley group than the maize and wheat groups. Compared to the other grain groups, the VW and VSA values were higher in the wheat group. The barley group had the highest CD, followed by the wheat and maize groups, and there were statistically significant differences among the groups. Broilers in the barley group had a substantially reduced VH/CD ratio compared to the other grain groups.

Although emulsifier supplementation of the reduced-energy diets did not change VH (*p* > 0.05), it significantly affected VW, CD, VH/CD, and VSA (*p* < 0.01). Compared with the standard-energy diets, the emulsifier-supplemented reduced-energy diets decreased jejunal VW, CD and VSA but increased the VH/CD ratio. The grain × emulsifier interaction significantly affected VW, CD, and VSA (*p* < 0.01), but not VH and VH/CD (*p* > 0.05). The VW of the maize with emulsifier group was lower than that of the other groups. The VW of the maize without emulsifier and wheat with/without emulsifier groups was higher than that of the maize with emulsifier and barley with/without emulsifier groups. The CD value of the barley without emulsifier group was significantly higher than that of the other groups, and the CD of the wheat without emulsifier group was significantly higher than that of all groups except for the barley without emulsifier group. The VSA value of the maize without emulsifier and wheat with/without emulsifier groups was significantly higher than that of the other groups.

## 4. Discussion

Feed consumption and feed passage rate through the digestive tract are positively correlated [29]. Grains contain water-soluble NSP, which are known to increase intestinal viscosity and thereby reduce feed consumption and passage rate [30]. In addition to this effect, cellulose present in the cell walls of grains such as barley acts as a physical barrier that encloses nutrients such as protein and carbohydrates, limiting their interaction with digestive enzymes [6]. In this study, broilers were fed identical nutrient-dense diets; however, feed consumption was lower in the wheat group, while the barley and maize groups consumed similar amounts. The body weights of broilers in the wheat group were also lower than those of broilers in the other grain groups. Feed efficiency was highest in the wheat group, followed by the maize and barley groups. Consistent with these findings, Kalantar et al. [31] reported that broilers fed diets containing wheat (15% or 20%) had lower feed consumption and body weight compared to those fed barley- and maize-based diets. According to Yaghobfar and Kalantar [32], wheat diets (15% or 20%) reduced feed consumption and, although inferior to maize in terms of body weight and feed efficiency, were similar to barley. In contrast, Rodríguez et al. [4] found that broilers fed barley-/wheat-based diets (30%/38.14%) had a lower body weight and feed consumption compared to maize. Similarly, Tang et al. [33] reported that broilers fed barley-based diets (65.2% or 66.9%) showed lower feed consumption, body weight, and feed efficiency than those fed maize- and wheat-based diets (60.5% or 64.7%). The reduced feed consumption in the wheat group may be attributed to increased intestinal viscosity caused by NSP, which slows gastric emptying [30]. In barley, the high cellulose content may have limited nutrient interaction with digestive fluids, but higher feed consumption compared to wheat partially compensated for this negative effect. Furthermore, the presence of an alpha-amylase inhibitor in wheat may have contributed to reduced body weight, as wheat—unlike barley and maize—impairs starch digestion [6,30]. Nevertheless, in this study, the feed conversion ratio was highest in barley and lowest in wheat, with maize being statistically similar to both and occupying an intermediate position. In a study examining the effects of maize, barley, and wheat on the feed conversion ratio in broilers, the lowest feed conversion ratio was obtained from maize-, wheat-, and barley-based diets, respectively. Kalantar et al. [31], Shakouri et al. [34] and Hashemi et al. [35] reported that feed consumption and feed efficiency were higher in maize-based diets than in wheat-barley-based diets. However, Rodríguez et al. [4] noted that different grains had no effect on the feed conversion ratio in broilers. dos Santos et al. [36] noted that birds fed barley-based diets performed better than those fed wheat-based diets. Such variable findings may be attributed to differences in bird genetics, grain type, and management practices. Emulsifiers are known to improve dietary fat digestibility, thereby increasing overall energy utilization [9,13,37]. In commercial practice, dietary energy is commonly reduced by approximately 50 kcal/kg when exogenous emulsifiers are used. Fayed et al. [38] demonstrated that their emulsifiers (250 g/ton) can effectively compensate for low dietary energy density (−80 kcal/kg) in broiler diets. Similar results have also been reported in studies where emulsifiers at levels of 750 or 800 g/ton were added to diets with a reduced ME of 60 or 100 kcal/kg [39,40]. These positive responses are primarily attributed to improved lipid metabolism and increased energy availability for tissue growth [41]. In this study, broiler performance did not differ between the emulsifier-supplemented reduced-energy diets and the standard-energy diets, which is consistent with the findings of Guerreiro Neto et al. [42], who reported no effect on performance at 42 days. Nevertheless, many previous studies have noted that emulsifiers increase body weight, reduce feed consumption, and improve feed efficiency [9,14,37,43,44,45]. Similar to Saleh et al. [46], these results indicate that emulsifier supplementation maintained broiler performance despite a 50 kcal/kg reduction in dietary ME. However, because the present design did not include a reduced-energy diet without emulsifier, the independent contributions of the emulsifier and the energy reduction to these responses cannot be separated.

Few studies have examined the effects of substituting barley or wheat for maize on carcass traits. Janocha et al. [47] reported that diets containing barley (30%) and wheat (29.3%) increased carcass yield, whereas Farran et al. [48] found no effect of barley inclusion on carcass characteristics. In this study, replacing maize with barley and wheat increased leg yield but reduced carcass and breast yield. Consistent with these findings, Bozkurt et al. [49] and Taheri and Shirzadegan [50] reported that wheat-based diets reduced nutrient digestibility and carcass yield compared to maize. Moharrery [51] noted that barley inclusion enhanced breast yield but did not affect carcass yield. Emulsifiers have been shown to promote muscle growth by enhancing lipid circulation [52]. In this study, emulsifier addition increased carcass yield, consistent with Kamel et al. [53]. However, several studies reported no effect of emulsifiers on carcass traits [14,37,42,43,44,45,46]. Notably, emulsifier supplementation significantly increased carcass yield in barley- and wheat-based diets but not in maize-based diets, suggesting that emulsifiers can enhance carcass yield when maize is replaced by barley or wheat without impairing performance.

Bone ash is a key indicator of bone quality, as higher ash content improves breaking strength. Tibia ash and breaking strength varied considerably with dietary grain source. Both parameters were highest in the wheat group and lowest in the barley group. In contrast, Pintar et al. [54] found no differences in tibia calcium and phosphorus among broilers fed maize, barley, or wheat. NSPs are known to impair mineral bioavailability [55], which may explain the low tibia ash in the barley group. Interestingly, tibia ash and strength were highest in the wheat group despite its high NSP content; this is possibly due to the lower body weight, as slower growth rates are associated with higher tibia ash and density [56]. Emulsifier supplementation increased tibia wall thickness and ash content. Although limited, studies have shown that emulsifiers increase tibia ash and mineral levels at 7 days of age, but not at 21 days [57]. Kamel et al. [53] reported significant increases in tibia calcium and phosphorus at 42 days with emulsifier supplementation. These effects may be explained by enhanced mineral absorption due to increased intestinal pore size [58,59], reduced saponification between fat and calcium [12], or stimulation of bone development [60]. In this study, emulsifiers increased tibia ash in wheat- and barley-based diets but not in maize-based diets. Interactions influenced wall thickness and tibia ash. The addition of emulsifier to diets comprising barley and wheat resulted in a rise in tibia ash; however, this effect was not seen in the group fed maize. It was revealed that tibia mineralization could not be further increased by supplementing with an emulsifier in birds fed maize-based diets but could be considerably increased in birds fed wheat- or barley-based diets.

Dietary composition and antinutritional factors influence digestive enzyme secretion and activity [61]. Water-soluble NSPs stimulate pancreatic secretions, particularly amylase, but exert a negative effect on pancreatic function [31,61]. Consistent with this, this study found that substituting maize with barley or wheat increased pancreatic amylase activity, which is in agreement with the findings of Kalantar et al. [31] and Yaghobfar and Kalantar [32]. NSP-induced viscosity increases endogenous secretions and nutrient loss [62], which aligns with the current findings. Emulsifiers, such as bile salts, enhance lipid hydrolysis by increasing micelle formation and lipase activity [8,63]. In this study, emulsifier supplementation increased pancreatic lipase activity, consistent with Guerreiro Neto et al. [42], though other studies reported no effect [13,64].

Maintaining jejunal histological health is critical for nutrient absorption. In this study, wheat improved jejunal villus morphology (except CD), barley reduced it, and maize showed intermediate effects. Shakouri et al. [34] similarly reported the lowest VH and VH/CD in barley-fed broilers. Kalantar et al. [31] and Yaghobfar and Kalantar [32] found that wheat and barley disrupted villus morphology, though the wheat effects differed from this study. The negative impact of barley may be linked to its higher cellulose content rather than NSP. Tejeda and Kim [65,66] confirmed that high dietary cellulose impairs intestinal morphology. Emulsifier supplementation improved the VH/CD ratio while reducing VW, CD, and VSA. These findings differ from Ahmadi-Sefat et al. [9], who reported no jejunal effects but noted changes in duodenal morphology. Other studies found no effect on ileal morphology [14,43,64]. This study suggests that emulsifiers promote villus development by reducing intestinal fermentation, supporting cell membranes, lowering inflammation, and enhancing short-chain fatty acid production [57,67,68,69,70,71].

## 5. Conclusions

Overall, the wheat-based diet reduced body weight and feed consumption, and pancreatic amylase activity was higher in birds fed barley- and wheat-based diets. Carcass yield was lower in broilers fed barley- and wheat-based diets than in those fed the maize-based diet, but this reduction was compensated for when these diets were fed as emulsifier-supplemented reduced-energy diets. Compared with the standard-energy diets, the emulsifier-supplemented reduced-energy diets (−50 kcal/kg ME) did not impair growth performance, increased tibia ash content in barley- and wheat-based diets, increased pancreatic lipase activity and improved the jejunal VH/CD ratio, although the other villus parameters decreased. These findings indicate that the response to emulsifier supplementation of reduced-energy diets depends on the grain source. Because the emulsifier supple-mentation and the energy reduction were applied together, these results reflect the combined feeding strategy rather than an isolated effect of the emulsifier. Further research, including reduced-energy diets without emulsifier and different emulsifier inclusion levels, is required to separate these effects.

## Figures and Tables

**Table 1:** Maize-based and emulsifier-added diets and nutrient contents.

Ingredients, g/kg	Maize			Maize + Emulsifier		
	Starter (0 to 10 d)	Grower (11 to 24 d)	Finisher (25 to 42 d)	Starter (0 to 10 d)	Grower (11 to 24 d)	Finisher (25 to 42 d)
Maize	486.9	533.3	591.2	499.9	544.3	602.2
Soybean meal	427.0	387.0	335.0	423.5	384.5	333.0
Soybean oil	49.0	50.0	47.5	39.0	41.0	38.0
Limestone	6.0	3.6	3.5	6.0	3.6	3.5
Dicalcium phosphate	23.0	19.0	15.8	23.0	19.0	15.8
Salt	2.5	2.5	2.5	2.5	2.5	2.5
Premix ^1^	1.0	1.0	1.0	1.0	1.0	1.0
L-lysine	2.0	1.3	1.5	2.0	1.3	1.5
DL-methionine	2.6	2.3	2.0	2.6	2.3	2.0
Emulsifier	0.0	0.0	0.0	0.5	0.5	0.5
Nutrients						
ME, kcal/kg	2977	3051	3106	2925	3004	3054
Crude protein, g/kg	230.5	215.4	195.5	230.0	215.1	195.3
Crude oil, g/kg	78.05	80.28	79.29	68.54	71.71	70.22
Crude fiber, g/kg	42.92	41.20	38.90	42.99	41.29	39.03
Calcium, g/kg	9.58	7.56	6.58	9.57	7.56	6.57
Total phosphorus, g/kg	8.63	7.77	7.00	8.65	7.78	7.01
Available phosphorus, g/kg	5.03	4.28	3.67	5.03	4.29	3.67
Lysine, g/kg	13.31	11.86	10.85	13.25	11.83	10.82
Methionine, g/kg	5.88	5.42	4.89	5.89	5.42	4.90
Methionine + cystine, g/kg	9.92	9.22	8.39	9.91	9.22	8.40
Soluble NSP, g/kg	5.55	5.26	4.89	5.55	5.27	4.89
Total NSP, g/kg	84.38	82.00	78.75	84.71	82.34	79.16

^1^ The premix provided the following per kg of diet: retinol acetate, 4.0 mg; cholecalciferol, 0.055 mg; DL-α-tocopheryl acetate, 11 mg; nicotinic acid, 44 mg; calcium-D-pantothenate, 8.8 mg; riboflavin sodium phosphate, 5.8 mg; thiamine hydrochloride, 2.8 mg; cyanocobalamin, 0.66 mg; folic acid, 1 mg; biotin, 0.11 mg; choline, 220 mg; Zn, 60 mg; Mn, 60 mg; Fe, 30 mg; Cu, 5 mg; I, 1.1 mg; Se, 0.1 mg.

**Table 2:** Barley-based and emulsifier-added diets and nutrient contents.

Ingredients, g/kg	Barley			Barley + Emulsifier		
	Starter (0 to 10 d)	Grower (11 to 24 d)	Finisher (25 to 42 d)	Starter (0 to 10 d)	Grower (11 to 24 d)	Finisher (25 to 42 d)
Maize	211.0	225.9	285.4	222.5	236.4	295.9
Barley	300.0	300.0	300.0	300.0	300.0	300.0
Soybean meal	338.0	368.0	316.0	335.5	366.5	314.0
Maize gluten	50.0	--	--	50.0	0.0	0.00
Soybean oil	62.5	77.0	73.0	53.0	67.5	64.0
Limestone	7.8	4.5	4.5	7.8	4.5	4.5
Dicalcium phosphate	21.0	17.5	14.0	21.0	17.5	14.0
Salt	2.5	2.5	2.5	2.5	2.5	2.5
Premix ^1^	1.0	1.0	1.0	1.0	1.0	1.0
L-lysine	3.7	1.4	1.6	3.7	1.4	1.6
DL-methionine	2.5	2.2	2.0	2.5	2.2	2.0
Emulsifier	0.0	0.0	0.0	0.5	0.5	0.5
Nutrients						
ME, kcal/kg	2977	3055	3101	2927	3002	3052
Crude protein, g/kg	230.3	215.1	195.3	230.1	215.1	195.2
Crude oil, g/kg	84.54	99.84	97.28	76.39	90.63	88.69
Crude fiber, g/kg	45.81	47.82	45.49	45.92	47.90	45.61
Calcium, g/kg	9.53	7.51	6.48	9.53	7.51	6.48
Total phosphorus, g/kg	8.18	7.62	6.79	8.20	7.63	6.81
Available phosphorus, g/kg	5.00	4.28	3.61	5.01	4.28	3.61
Lysine, g/kg	13.30	11.87	10.86	13.26	11.86	10.83
Methionine, g/kg	6.01	5.24	4.81	6.01	5.25	4.81
Methionine + cystine, g/kg	9.64	9.06	8.34	9.64	9.08	8.35
Soluble NSP, g/kg	15.76	15.86	15.48	15.77	15.86	15.48
Total NSP, g/kg	111.38	110.34	107.00	111.75	110.59	107.38

^1^ The premix provided the following per kg of diet: retinol acetate, 4.0 mg; cholecalciferol, 0.055 mg; DL-α-tocopheryl acetate, 11 mg; nicotinic acid, 44 mg; calcium-D-pantothenate, 8.8 mg; riboflavin sodium phosphate, 5.8 mg; thiamine hydrochloride, 2.8 mg; cyanocobalamin, 0.66 mg; folic acid, 1 mg; biotin, 0.11 mg; choline, 220 mg; Zn, 60 mg; Mn, 60 mg; Fe, 30 mg; Cu, 5 mg; I, 1.1 mg; Se, 0.1 mg.

**Table 3:** Wheat-based and emulsifier-added diets and nutrient contents.

Ingredients, g/kg	Wheat			Wheat + Emulsifier		
	Starter (0 to 10 d)	Grower (11 to 24 d)	Finisher (25 to 42 d)	Starter (0 to 10 d)	Grower (11 to 24 d)	Finisher (25 to 42 d)
Wheat	540.2	561.2	624.5	552.1	573.2	635.5
Soybean meal	331.0	346.0	289.0	327.5	343.0	286.0
Maize gluten	40.0	0.0	0.0	40.0	0.0	0.0
Soybean oil	50.0	63.0	60.0	41.0	53.5	51.5
Limestone	7.7	4.7	4.8	7.7	4.7	4.8
Dicalcium phosphate	21.3	17.5	14.0	21.3	17.5	14.0
Salt	2.5	2.5	2.5	2.5	2.5	2.5
Premix ^1^	1.0	1.0	1.0	1.0	1.0	1.0
L-lysine	3.8	1.9	2.2	3.9	1.9	2.2
DL-methionine	2.5	2.2	2.0	2.5	2.2	2.0
Emulsifier	0.0	0.0	0.0	0.5	0.5	0.5
Nutrients						
ME, kcal/kg	2975	3055	3100	2926	3003	3052
Crude protein, g/kg	230.3	215.3	195.4	230.0	215.3	195.1
Crude oil, g/kg	65.55	77.99	74.86	56.66	68.62	66.47
Crude fiber, g/kg	36.74	37.82	35.17	36.76	37.88	35.21
Calcium, g/kg	9.53	7.50	6.50	9.53	7.50	6.50
Total phosphorus, g/kg	7.96	7.29	6.45	7.98	7.30	6.46
Available phosphorus, g/kg	5.01	4.25	3.61	5.02	4.26	3.62
Lysine, g/kg	13.25	11.87	10.88	13.28	11.84	10.84
Methionine, g/kg	5.99	5.23	4.85	5.99	5.28	4.85
Methionine + cystine, g/kg	9.84	9.25	8.54	9.85	9.26	8.55
Soluble NSP, g/kg	10.50	10.72	10.96	10.62	10.85	11.07
Total NSP, g/kg	95.16	94.14	92.36	95.75	94.81	92.94

^1^ The premix provided the following per kg of diet: retinol acetate, 4.0 mg; cholecalciferol, 0.055 mg; DL-α-tocopheryl acetate, 11 mg; nicotinic acid, 44 mg; calcium-D-pantothenate, 8.8 mg; riboflavin sodium phosphate, 5.8 mg; thiamine hydrochloride, 2.8 mg; cyanocobalamin, 0.66 mg; folic acid, 1 mg; biotin, 0.11 mg; choline, 220 mg; Zn, 60 mg; Mn, 60 mg; Fe, 30 mg; Cu, 5 mg; I, 1.1 mg; Se, 0.1 mg.

**Table 4:** Effects of grain source and emulsifier supplementation of reduced-energy diets on the performance of broilers.

Grain	Emulsifier	Hatching Body Weight, g	Body Weight, g	Body Weight Gain, g	Feed Consumption, g/bird	Feed Conversion Ratio, feed/gain
Maize		42.59	2711 ^A^	2669 ^A^	4348 ^A^	1.629 ^ab^
Barley		43.02	2683 ^A^	2641 ^A^	4336 ^A^	1.642 ^a^
Wheat		42.73	2543 ^B^	2500 ^B^	4030 ^B^	1.612 ^b^
SEM		0.276	37.23	37.32	54.46	0.0065
	0	42.70	2670	2627	4271	1.626
	500	42.86	2622	2579	4205	1.630
	SEM	0.232	34.15	35.21	57.78	0.0061
Maize	0	42.46	2706	2664	4310	1.619
Maize	500	42.73	2716	2674	4386	1.640
Barley	0	43.25	2725	2682	4422	1.649
Barley	500	42.79	2642	2599	4250	1.636
Wheat	0	42.40	2577	2535	4081	1.610
Wheat	500	43.06	2508	2465	3980	1.615
SEM		0.932	50.57	50.67	72.01	0.0082
*p*-values						
Grain		0.582	0.010	0.010	<0.001	0.011
Emulsifier		0.644	0.290	0.289	0.300	0.586
Grain × Emulsifier		0.404	0.649	0.652	0.261	0.175

^A,B^ Differences between means shown with different letters in the same column are statistically significant at the *p* < 0.01 level. ^a,b^ Differences between means shown with different letters in the same column are statistically significant at the *p* < 0.05 level.

**Table 5:** Effects of grain source and emulsifier supplementation of reduced-energy diets on the slaughtering traits of broilers.

Grain	Emulsifier, g/ton	Carcass, % of Body Weight	Breast, % of Carcass	Legs, % of Carcass	Abdominal Fat, % of Body Weight	Liver, % of Body Weight	Gizzard, % of Body Weight
Maize		75.25 ^A^	37.79 ^A^	28.02 ^B^	0.963	1.672	1.409
Barley		74.14 ^B^	35.18 ^B^	28.87 ^AB^	0.934	1.611	1.475
Wheat		73.79 ^B^	34.10 ^B^	29.57 ^A^	0.827	1.694	1.381
SEM		0.367	0.340	0.299	0.0581	0.0341	0.0438
	0	73.69 ^B^	35.49	28.87	0.905	1.677	1.464
	500	75.09 ^A^	35.89	28.77	0.910	1.642	1.380
	SEM	0.341	0.510	0.291	0.0490	0.0290	0.0370
Maize	0	75.36 ^A^	37.80	27.59	0.923	1.668	1.396
Maize	500	75.14 ^A^	37.77	28.46	1.003	1.676	1.423
Barley	0	72.92 ^B^	35.07	29.34	0.883	1.639	1.582
Barley	500	75.35 ^A^	35.30	28.40	0.985	1.584	1.368
Wheat	0	72.80 ^B^	33.61	29.68	0.910	1.724	1.413
Wheat	500	74.78 ^A^	34.60	29.45	0.743	1.665	1.350
SEM		0.327	0.493	0.374	0.0742	0.0478	0.0555
*p*-values							
Grain		0.001	<0.001	0.004	0.245	0.259	0.289
Emulsifier		<0.001	0.400	0.772	0.942	0.405	0.096
Grain ×Emulsifier		0.002	0.658	0.109	0.220	0.762	0.145

^A,B^ Differences between means shown with different letters in the same column are statistically significant at the *p* < 0.01 level.

**Table 6:** Effects of grain source and emulsifier supplementation of reduced-energy diets on the tibia traits of broilers.

Grain	Emulsifier, g/ton	Weight, g	Length, mm	Diameter, mm	Wall Thickness, mm	Breaking Strength, N	Ash, %
Maize		18.37	106.61	9.15	1.69	330.21 ^B^	41.63 ^B^
Barley		18.83	106.93	9.49	1.74	309.29 ^C^	41.07 ^C^
Wheat		18.86	106.38	9.56	1.81	346.41 ^A^	42.15 ^A^
SEM		0.327	0.300	0.143	0.0542	2.086	0.239
	0	18.40	106.53	9.24	1.68 ^B^	326.02	41.40 ^B^
	500	18.98	106.75	9.56	1.81 ^A^	330.59	41.83 ^A^
	SEM	0.265	0.244	0.126	0.0508	4.445	0.079
Maize	0	18.28	106.84	8.80	1.65 ^BC^	327.43	41.30 ^B^
Maize	500	18.47	106.38	9.51	1.74 ^BC^	333.00	41.96 ^B^
Barley	0	18.92	106.96	9.48	1.83 ^B^	306.85	40.12 ^C^
Barley	500	18.75	106.91	9.49	1.64 ^BC^	309.72	42.02 ^B^
Wheat	0	17.99	105.80	9.45	1.57 ^C^	343.77	41.51 ^B^
Wheat	500	19.73	106.95	9.67	2.06 ^A^	349.04	42.79 ^A^
SEM		0.413	0.395	0.183	0.0449	2.598	0.157
*p*-values							
Grain		0.442	0.412	0.106	0.077	<0.001	<0.001
Emulsifier		0.103	0.527	0.058	0.006	0.078	0.006
Grain × Emulsifier		0.076	0.150	0.205	<0.001	0.889	<0.001

^A,B,C^ Differences between means shown with different letters in the same column are statistically significant at the *p* < 0.01 level.

**Table 7:** Effects of grain source and emulsifier supplementation of reduced-energy diets on the pancreas parameters of broilers.

Grain	Emulsifier, g/ton	Pancreas, % of Body Weight	Lipase, U/mg	Amylase, U/mg	Protease, U/mg
Maize		0.256	1.213	0.331 ^B^	2.910
Barley		0.251	1.153	0.666 ^A^	3.532
Wheat		0.251	1.157	0.609 ^A^	3.341
SEM		0.0132	0.1720	0.0798	0.6591
	0	0.261	0.807 ^B^	0.513	3.326
	500	0.245	1.541 ^A^	0.544	3.196
	SEM	0.0109	0.1210	0.0787	0.5337
Maize	0	0.246	0.948 ^bc^	0.240	3.002
Maize	500	0.267	1.478 ^ab^	0.383	2.818
Barley	0	0.279	1.024 ^abc^	0.811	3.919
Barley	500	0.222	1.282 ^abc^	0.520	3.146
Wheat	0	0.257	0.450 ^c^	0.489	3.058
Wheat	500	0.245	1.864 ^a^	0.730	3.624
SEM		0.0167	0.1611	0.0988	0.9703
*p*-values					
Grain		0.942	0.945	0.009	0.811
Emulsifier		0.313	<0.001	0.738	0.872
Grain × Emulsifier		0.129	0.020	0.058	0.793

^A,B^ Differences between means shown with different letters in the same column are statistically significant at the *p* < 0.01 level. ^a,b,c^ Differences between means shown with different letters in the same column are statistically significant at the *p* < 0.05 level.

**Table 8:** Effects of grain source and emulsifier supplementation of reduced-energy diets on the jejunum histomorphology of broilers.

Grain	Emulsifier, g/ton	Villus Height, µm	Villus Width, µm	Crypt Depth, µm	Villus Height/Crypt Depth	Villus Surface Area, mm^2^
Maize		810 ^B^	98.7 ^B^	154.6 ^C^	5.42 ^A^	0.249 ^B^
Barley		773 ^C^	97.6 ^B^	180.0 ^A^	4.48 ^B^	0.238 ^B^
Wheat		861 ^A^	107.7 ^A^	169.0 ^B^	5.48 ^A^	0.290 ^A^
SEM		26.9	4.16	8.16	0.307	0.0143
	0	806	106.0 ^A^	179.8 ^A^	4.80 ^B^	0.268 ^A^
	500	824	96.7 ^B^	156.0 ^B^	5.45 ^A^	0.250 ^B^
	SEM	14.8	2.28	11.27	0.174	0.0082
Maize	0	791	110.4 ^A^	159.3 ^C^	5.11	0.274 ^A^
Maize	500	829	86.9 ^C^	149.9 ^C^	5.72	0.224 ^B^
Barley	0	782	99.5 ^B^	199.2 ^A^	4.04	0.245 ^B^
Barley	500	765	95.8 ^B^	160.8 ^C^	4.92	0.230 ^B^
Wheat	0	846	108.1 ^A^	180.8 ^B^	5.24	0.285 ^A^
Wheat	500	877	107.5 ^A^	157.2 ^C^	5.72	0.295 ^A^
SEM		73.8	11.39	23.81	0.890	0.0398
*p*-values						
Grain		<0.001	<0.001	<0.001	<0.001	<0.001
Emulsifier		0.102	<0.001	<0.001	<0.001	0.001
Grain ×Emulsifier		0.061	<0.001	<0.001	0.385	<0.001

^A,B,C^ Differences between means shown with different letters in the same column are statistically significant at the *p* < 0.01 level.

## Data Availability

The data presented in this study are available upon request from the corresponding author.
